# Stable carbon isotopes of woods during carbonization and their correlation with climatic factors

**DOI:** 10.1371/journal.pone.0270133

**Published:** 2022-10-20

**Authors:** Fan Luo, Nan Sun, Xiabo Li, Junfeng Guo, Liang Xiao, Peng Lei

**Affiliations:** 1 School of Earth Science and Resources, Chang’an University, Xi’an, Shaanxi, China; 2 Shaanxi Key Laboratory of Early Life and Environments, Northwest University, Xi’an, Shaanxi, China; 3 Shaanxi Branch of China National Geological Exploration Center of Building Materials Industry, Xi’an, Shaanxi, China; Institute of Earth and Environment, Chinese Academy of Sciences, CHINA

## Abstract

To explore the applicability of the carbon isotope composition (δ^13^C) of fossil charcoal for the quantitative reconstruction of paleoclimates, we selected five points in Shaanxi province, from north to south, to collect modern *Pinus* species and *Quercus* species to sample covering areas with obvious climatic differences. In order to reveal the relationships between δ^13^C of charcoal and climate variables on the basis of carbonization experiments, we evaluated the fractionation mechanism of δ^13^C of charcoal, and compared the differences between δ^13^C of charcoal in wildfire experiments and indoor experiments regarding genera and species. The results showed significant differences in δ^13^C between genera but no significant differences among species. Additionally, the δ^13^C of charcoal was significantly negatively correlated with precipitation and positively correlated with evaporation, which could be determined from δ^13^C values in the study area to reconstruct ancient precipitation and evaporation in the future.

## 1. Introduction

Climate has attracted wide attention from the international community and academic circles as an important factor affecting the human living environment [1–3]. The period 2010–2019 is the hottest decade since modern meteorological observations were recorded [4]. Global warming has become an indisputable fact, and the warming effect has become a hot topic of interest in the international community [5]. Understanding the laws governing climate change and its warming effect is a key problem to be solved. The high–resolution quantitative reconstruction of paleoclimate plays an important role in understanding the changes in climate systems.

In recent years, the quantitative study of plant indicators has produced many results, such as the hydrological and climatic trends of lakes in the southwestern part of the Loop Basin since 40 ka, which were quantitatively reconstructed by several organic matter geochemical indicators [6]. By establishing the magnetization rate of loess-paleosoil as a function of mean annual rainfall, we recovered the paleosoil rainfall during the warm interglacial period of the Loess Plateau [7]. By establishing the transfer function of diatom-climate factors, we provided a basis for paleoclimate reconstruction at different scales in southwestern China [8]. By the numerical conversion function of lake mesocosms and temperature, we quantitatively reconstructed the Late Pleistocene temperature peaks in central Italy by the numerical conversion function between lake mesocosms and temperature [9]. Using carbonate sediments in lakes, we quantitatively reconstructed the early to mid-Holocene hydrological climate change in eastern Washington [10], and the Late Holocene rainfall at Karwar, west coast of India, was quantitatively reconstructed by a conversion function of the sporulation diversity to climate factors [11]. Based on a phytolith-climate factor conversion function, the annual average temperature, rainfall, and relative humidity in the peat section of an elm tree in Northeast China was inferred for the past three thousand years [12]. Temperature and rainfall variations between 6200 yr. cal BP-5600 yr. cal BP in the Guanzhong Basin of the southern Loess Plateau were quantitatively reconstructed by charcoal [13,14]. However, in the past, paleoclimate reconstruction using plant indicators mainly used the coexistence approach or the transformation function method. The accuracy of the former depends on the number of sample species; the more species considered, the higher the accuracy [15]. If the number of species obtained at the sampling site is small, the error of the obtained results is large. The use of the latter is based on the premise that plant indicators are linearly related to climate, and the number of species is required to be sufficiently large, with limited in its application [16]. Therefore, more accurate and better reconstruction methods are needed.

In response, an isotopic analysis method has been proposed [17–19], and the application of this method to pollen [20–22], phytoliths [23–25], and seeds [26] have revealed a new opportunity for high-precision quantitative reconstruction of paleoclimates. Tree annual rings with good temporal continuity and high accuracy have long been important indicators for paleoclimate studies, and recent studies on tree wheel isotopes have greatly improved the accuracy of paleoclimate reconstruction [27–34]. The analysis of charcoal, as plant fossils, is an important indicator for paleoclimate reconstruction due to its easy accessibility and high identification accuracy [19,30,35–37], especially in areas where sporulation is poorly preserved. Charcoal remains have become an important complement to sporulation studies, and thus, the paleoclimatic significance of charcoal isotopes has become a hot topic of interest for scholars.

Recent studies have shown a significant correlation between charcoal stable carbon isotopes and climate factors [38,39]. Accordingly, the results of studying carbon isotopes in charcoal at Neanderthal sites from the Paleolithic Age showed that the climate in the study area changed periodically from humid to arid [37]. The results of charcoal isotope studies at the Sibudu Cave site indicate that the study area underwent a warm-wet to cold-dry to warm-wet variation between 70 ka and 48 ka [35]. The above-mentioned studies qualitatively reconstructed the paleoclimate through charcoal stable isotopes with low precision; therefore, some scholars reconstructed the paleoclimate quantitatively by establishing isotope-climate factor relationship functions. For example, the analysis of charcoal carbon isotopes in the archaeological sites of the Segre and Cinca Valleys concluded that the climate of the Iberian Peninsula from 2000 B.C. to A.D. For example, the driest period in the Iberian Peninsula from 2000 B.C.E. to A.D. from 900 to 300 B.C.E. [19], and charcoal carbon isotope analysis of the archaeological site of Valencia Province showed that the climate changed throughout the Holocene in the study area, and the precipitation in the spring and summer of B.C.E. was always higher than the present [40]. The above findings provide new ideas for the quantitative reconstruction of paleoclimate, but the following questions remain to be explored:

1. The climate-charcoal δ^13^C model in the previous study was based on the carbonization of the present species in a muffle furnace, while the charcoal at the site was formed by wildfire.
2. The climate-charcoal δ^13^C models in previous studies were based on the species level, but since charcoal is often identified only at the genus level and a few species can be identified at the species level, is it possible to model δ^13^C at the genus level?
3. Previous work has mainly focused on Europe, America and Africa, and the data from the above research cannot be directly applied to other regions due to differences in environment and species distribution in different regions.

To solve the above problems, improve the application of stable carbon isotopes in quantitative paleoclimate reconstruction, and obtain more accurate paleoclimate data, we selected areas sensitive to climate change and rich in charcoal remains for our research work. By collecting modern wood, conducting wildfire and laboratory carbonization experiments to compare their differences, determining the mechanism of stable carbon isotope fractionation in wood, comparing the variability of stable carbon isotope fractionation between genera and species, and exploring the correlation between charcoal stable carbon isotopes and climatic factors, we applied the method to archeological sites and obtained accurate paleoclimatic information in the study area.

## 2. Study area

Shaanxi Province, which has a continental monsoon climate overall, is composed of three geomorphologic units located on the northern Loess Plateau, in the central Guanzhong Basin and in the southern Shannan Mountains, corresponding to the middle temperate, warm temperate and subtropical climatic zones, respectively. The average annual rainfall and temperature gradually increase from north to south. The average annual temperature of northern Shaanxi is 7–12°C, where the average annual rainfall is 472 mm; the corresponding values for Guanzhong are 12–14°C and 504 mm, and those for southern Shaanxi are approximately 13–15°C and 1276 mm, respectively (http://data.cma.cn/). Rainfall is mainly concentrated in summer and autumn.

### 3. Materials and methods

#### 3.1 Sampling

Through the statistical analysis of the types of charcoal species at the existing archeological sites, it was found that *Pinus* sp. and *Quercus* sp. are the two most common wood types at these sites [41–43]. *Pinus* sp. is distributed from the Northern Hemisphere in the Arctic through North Africa, Central America and South Asia to south of the equator. There are 22 *Pinus* species in China. *Quercus* sp. is distributed in the northern temperate zone and tropical mountains, and there are approximately 110 species in China, distributed in both northern and southern provinces. There *Pinus* species and five *Quercus* species were examined in this study, which is useful for investigating the differences in stable isotopes among species.

**Fig 1:**
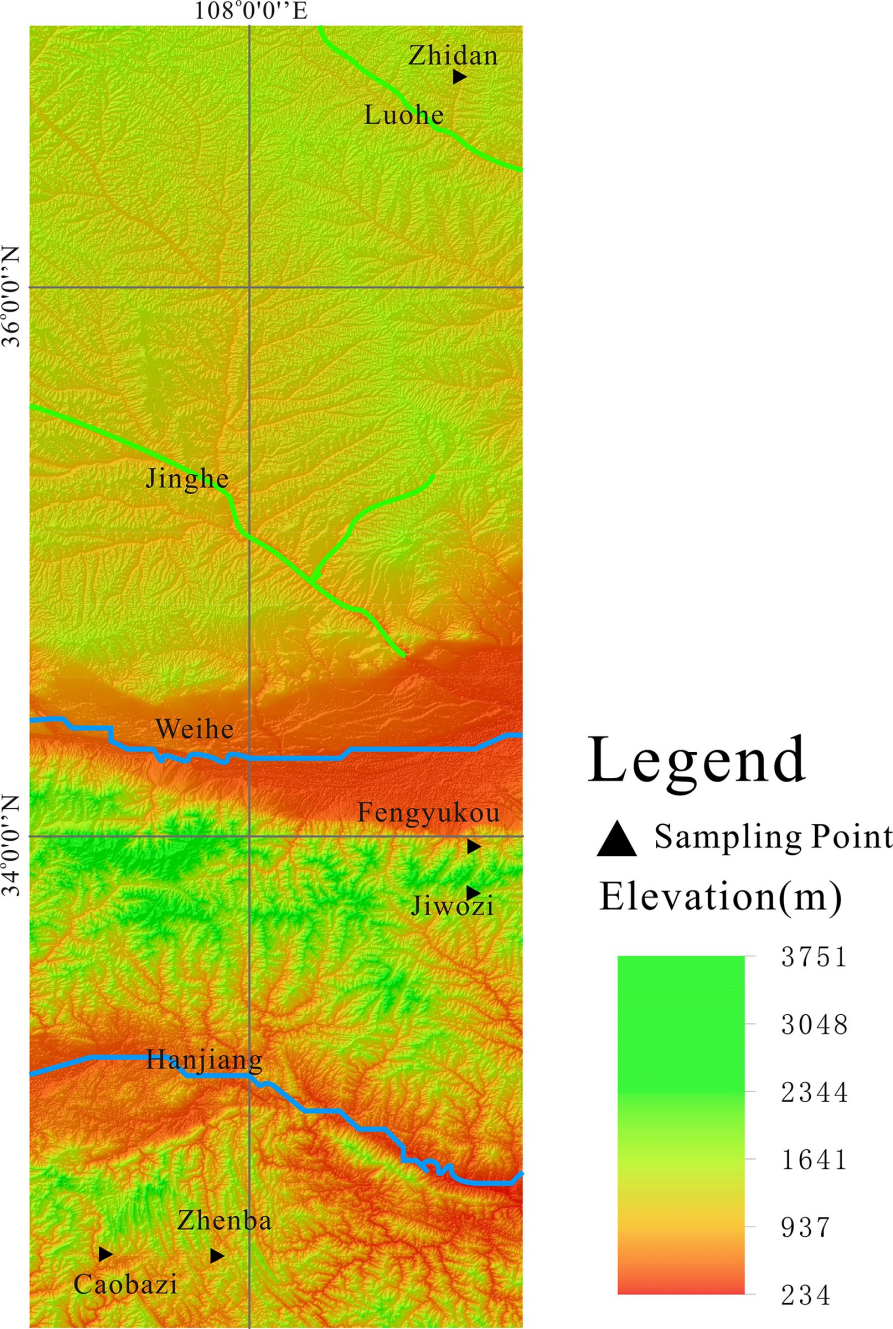
Map of the sampling sites. (http://landsat.visibleearth.nasa.gov/)[44].

In this study, two plant samples of *Pinus* sp. and *Quercus* sp. were collected in five regions (Fig 1) of Zhidan County, Fengyukou, Jiwozi, Zhenba County and Caobazi (Table 1). A total of 12 trees were sampled, and uniform samples were selected from small tree branches with a diameter of 5 cm.

**Table 1:** Geographic and climatic parameters of the sampling sites (http://www.geodata.cn) [45].

Site	Jiwozi	Caobazi	Zhenba	Fengyukou	Zhidan
Longitude	108°49′29.64″	107°29′6.72″	107°53′29.76″	108°49′39.36″	108°46′33.24″
Latitude	33°51′11.16″	32°32′16.08″	32°31′52.68″	34°1′24.96″	36°49′41.16″
Temperature(°C)	6.8	9.98	13.78	13.36	9.4
Evaporation(mm)	829.08	914.16	1060.8	1062.24	1088.28
Preciptation(mm)	1424.88	1374.6	1009.8	711.6	489.6
Genus	*Pinus*(*Pinus massoniana*)	*Pinus*(*Pinus tabulaeformis*)	*Pinus*(*Pinus massoniana; Pinus armandii*)	*Pinus*(*Pinus tabulaeformis*)	*Pinus*(*Pinus tabulaeformis*)
		*Quercus*(*Quercus acutissima Carr*)	*Quercus*(*Quercus aliena Blume* var.)	*Quercus*(*Quercus dentate Thunb*.; *Quercus aliena Blume*; *Quercus acutissima Carr*)	*Quercus*(*Quercus mongolica Fischer*)
				

### 3.2 Experimental carbonization

After the collected samples were dried naturally, they were cut into cylinders of 1 cm. Samples were cut from each collected branch and divided into five groups. Each group was composed of two samples. One of them was used as a control group, in which no carbonization treatment was carried out, and the remaining four pieces were wrapped with aluminum foil. Three of these four pieces were used in indoor experiments, and the last was used in a wildfire experiment.

The three samples wrapped in aluminum foil were placed in a muffle furnace and carbonized at temperatures of 300°C, 400°C and 500°C for 30 min; this combustion was allowed to continue for 240 min [46,47].

The last sample wrapped in aluminum foil was placed next to a fire ignited in an open space so that it would be completely carbonized.

### 3.3 Stable isotope determination

All samples were ground into powder with a mortar to stabilize the determination of carbon isotopes. In this experiment, the classic closed bottle method of organic carbon isotope analysis was adopted. An appropriate amount of the sample was mixed with copper oxide, platinum wire and copper wire and placed in a quartz bottle. The bottle was first subjected to vacuum pumping with a vacuum line. Thereafter, the bottle was removed and placed in a muffle furnace, where oxidation was conducted at 850°C for 2.5 hours.

The CO_2_ gas was collected and purified with a liquid nitrogen cold trap and then sent to a mass spectrometer for carbon isotope analysis. The instrument used in the experiment was a MAT251 stable isotope mass spectrometer from the Institute of Earth Environment, Chinese Academy of Sciences. The analytical accuracy corresponded to the national standard furnace black (GBW04407) carbon isotope analysis accuracy, which was better than ±0.1‰.

### 3.4 Analysis methods

In this study, statistical analysis of the data was performed using Origin and SPSS software. Since the distribution type of the samples was unknown, the widely applied independent sample nonparametric test was used to compare the similarities and differences of charcoal δ^13^C between genera and species and the similarities and differences of fractionation results of different carbonization methods; then, principal component analysis and correlation analysis were performed on charcoal δ^13^C with rainfall, evaporation and temperature to reveal the correlation between δ^13^C and rainfall, evaporation and temperature; finally, the linear fitting method was used. Finally, a linear regression was performed on the climate factors significantly correlated with charcoal δ^13^C to establish the charcoal δ^13^C-climate factor relationship function.

## 4. Results

The samples were analyzed via stable carbon analysis, and the results are shown in Table 2. In Caobazi, without carbonization, the range of δ^13^C values in *Pinus* sp. was –27.33‰ to –27.49‰, and the range of δ^13^C values in *Quercus* sp. was –29.14‰ to –29.46‰. Under the conditions of carbonization at 300°C, the range of δ^13^C values in *Pinus* sp. was –28.23‰ to –28.38‰, and that in *Quercus* sp. was –29.49‰ to –29.86‰. Under the conditions of carbonization at 400°C, the range of δ^13^C values in *Pinus* sp. was –28.21‰ to –28.21‰, and that in *Quercus* sp. was –29.80‰ to –29.92‰. Under the conditions of carbonization at 500°C, the range of δ^13^C values in *Pinus* sp. was –28.35‰ to –28.45‰, and that in *Quercus* sp. was –29.20‰ to –29.20‰. Under the conditions of carbonization by wildfire, the range of δ^13^C values in *Pinus* sp. was –28.42‰ to –28.42‰, and that in *Quercus* sp. was –29.99‰ to –31.66‰.

**Table 2:** δ^13^C (‰ VPDB) of wood samples heated at different temperatures.

Site	Genus	Untreated 300°C 400°C 500°C wild
Zhidan	*Pinus tabulaeformis* *Quercus mongolica Fischer*	-26.07 -26.34 -26.14 -27.07 -26.18 -25.58 -26.13 -26.49 -26.57 -28.07 -26.49 -26.74 -26.87 -26.89 -27.62 -26.48 -26.60 -26.83 -27.41 -27.69
Fengyukou	*Pinus tabulaeformis* *Quercus acutissima Carr* *Quercus aliena Blume* *Quercus dentata Thunb*	-25.83 -25.77 -26.35 -26.95 -26.82 -25.37 -26.40 -26.58 -26.89 -25.88 -28.10 -28.35 -27.26 -28.41 -27.91 -27.92 -27.70 -27.58 -27.82 -27.91 -27.52 -27.95 -28.32 -27.92 -27.86 -27.44 -28.23 -28.47 -27.67 -27.66 -27.27 -27.98 -28.01 -26.98 -28.30 -27.24 -27.60 -26.79 -27.44 -27.70
Zhenba	*Pinusmassoniana* *Pinus armandii* *Quercus aliena Blume var*.	-26.92 -27.46 -28.12 -27.33 -27.62 -26.84 -27.50 -28.04 -27.72 -27.52 -26.74 -27.55 -27.77 -28.21 -26.84 -26.77 -27.57 -28.28 -28.05 -28.14 -27.87 -27.91 -28.35 -28.33 -28.96 -28.09 -28.35 -28.37 -28.19 -29.06
Caobazi	*Pinus tabulaeformis* *Quercus acutissima Carr*	-27.33 -28.23 -28.21 -28.45 -28.42 -27.49 -28.38 -28.21 -28.35 -28.42 -29.14 -29.49 -29.80 -29.20 -31.66 -29.46 -29.86 -29.92 -29.20 -29.99
Jiwozi	*Pinusmassoniana*	-28.49 -29.62 -29.36 -29.45 -29.82 -28.66 -29.02 -29.46 -29.56 -29.71

In Zhenba, without carbonization, the range of δ^13^C values in *Pinus* sp. was –26.74‰ to –26.92‰, and the range of δ^13^C values in *Quercus* sp. was –27.87‰ to –28.09‰. Under the conditions of carbonization at 300°C, the range of δ^13^C values in *Pinus* sp. was –27.46‰ to –27.57‰, and that in *Quercus* sp. was –27.91‰ to –28.35‰. Under the conditions of carbonization at 400°C, the range of δ^13^C values in *Pinus* sp. was –27.77‰ to –28.28‰, and that in *Quercus* sp. was –28.35‰ to –28.37‰. Under the conditions of carbonization at 500°C, the range of δ^13^C values in *Pinus* sp. was –27.33‰ to –28.21‰, and that in *Quercus* sp. was –28.19‰ to –28.33‰. Under the conditions of carbonization by wildfire, the range of δ^13^C values in *Pinus* sp. was –26.84‰ to –28.14‰, and that in *Quercus* sp. was –28.96‰ to –29.06‰.

In Jiwozi, without carbonization, the range of δ^13^C values in *Pinus* sp. was –28.49‰ to –28.66‰. Under the conditions of carbonization at 300°C, the range of δ^13^C values in *Pinus* sp. was –29.02‰ to –29.62‰. Under the conditions of carbonization at 400°C, the range of δ^13^C values in *Pinus* sp. was –29.36‰ to –29.46‰. Under the conditions of carbonization at 500°C, the range of δ^13^C values in *Pinus* sp. was –29.45‰ to –29.56‰. Under the conditions of carbonization by wildfire, the range of δ^13^C values in *Pinus* sp. was –29.71‰ to –29.82‰.

In Fengyukou without carbonization, the range of δ^13^C values in *Pinus* sp. was –25.37‰ to –25.83‰, and that in *Quercus* sp. was –27.24‰ to –28.10‰. Under the conditions of carbonization at 300°C, the range of δ^13^C values in *Pinus* sp. was –25.77‰ to –26.40‰, and that in *Quercus* sp. was –27.60‰ to –28.35‰. Under the conditions of carbonization at 400°C, the range of δ^13^C values in *Pinus* sp. was –26.35‰ to –26.58‰, and that in *Quercus* sp. was –26.79‰ to –28.47‰. Under the conditions of carbonization at 500°C, the range of δ^13^C values in *Pinus* sp. was –26.89‰ to –26.95‰, and that in *Quercus* sp. was –26.98‰ to –28.41‰. Under the conditions of carbonization by wildfire, the range of δ^13^C values in *Pinus* sp. was –25.88‰ to –26.82‰, and that in *Quercus* sp. was –27.66‰ to –28.30‰.

In Zhidan, without carbonization, the range of δ^13^C values in *Pinus* sp. was –25.58‰ to –26.07‰, and that in *Quercus* sp. was –26.48‰ to –26.49‰. Under the conditions of carbonization at 300°C, the range of δ^13^C values in *Pinus* sp. was –26.13‰ to –26.34‰ and that in *Quercus* sp. was –26.60‰ to –26.88‰. Under the conditions of carbonization at 400°C, the range of δ^13^C values in *Pinus* sp. was –26.14‰ to –26.49‰, and that in *Quercus* sp. was –26.83‰ to –26.87‰. Under the conditions of carbonization at 500°C, the range of δ^13^C values in *Pinus* sp. was –26.57‰ to –27.07‰, and that in *Quercus* sp. was –26.89‰ to –27.41‰. Under the conditions of carbonization by wildfire, the range of δ^13^C values in *Pinus* sp. was –26.18‰ to –28.07‰, and that in *Quercus* sp. was –27.62‰ to -27.69‰.

According to our experimental data (Table 2), the δ^13^C values of different genera differed, and the values for the same genus varied in diverse growth environments. Under various carbonization conditions, the δ^13^C is most positive at Jiwozi among five sites.

## 5. Discussion

### 5.1 Characteristics of stable carbon isotopes in different genera and species after wood carbonization

**Fig 2:**
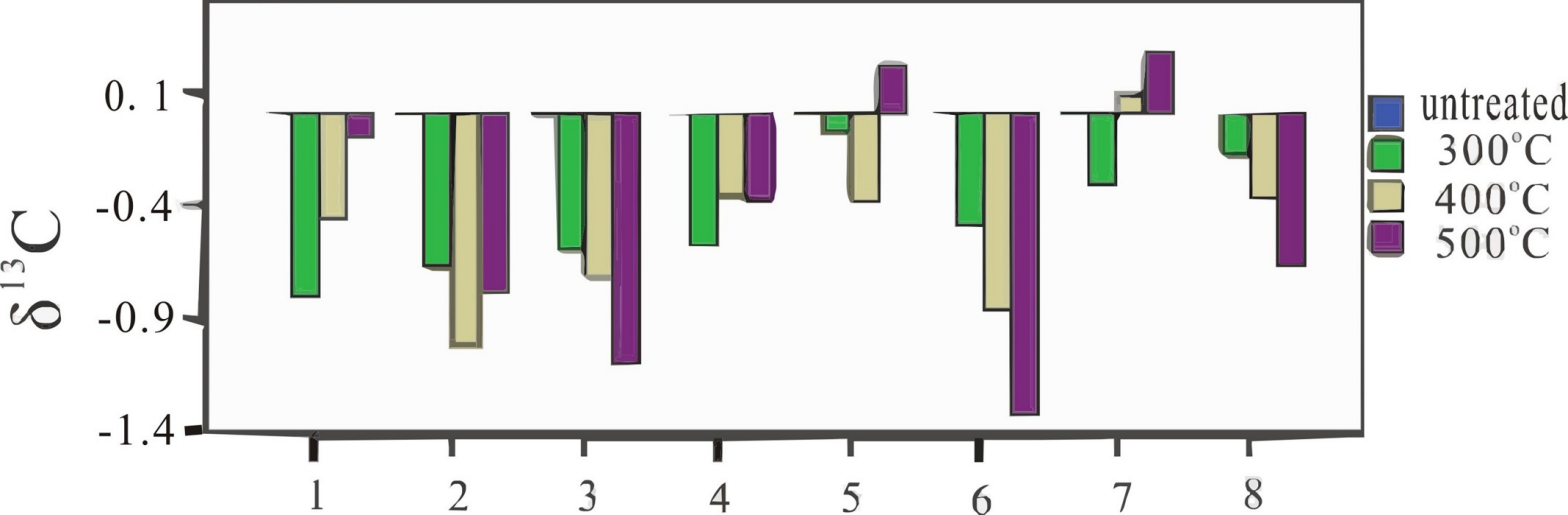
δ^13^C (‰ VPDB) mean fractionation values of wood samples of *Pinus* sp. and *Quercus* sp. at different temperatures. (δ^13^C mean fractionation values of untreated wood is zero. 1- *Pinus armandii*; 2- *Pinus massoniana*; 3- *Pinus tabulaeformis*; 4- *Quercus acutissima Carr*; 5- *Quercus aliena Blume*; 6- *Quercus aliena Blume* var.; 7- *Quercus dentata Thunb*; 8- *Quercus mongolica Fischer*).

Previous studies have shown that the pyrolysis of wood hemicellulose, cellulose and lignin occurs at 170–240°C, 240–310°C, and 320–400°C, respectively, during the heating process [48]. At temperatures above 400°C, the carbon-containing organic matter of wood is basically completely pyrolyzed [2,46] and forms C = C bonds with a stable structure [49,50]. Hemicellulose shows the highest δ^13^C values in wood, cellulose shows the second highest, and lignin shows the lowest [17,18,51]. Therefore, with increasing carbonization temperature, the δ^13^C value of charcoal decreases gradually, and the δ^13^C values of charcoal decrease substantially from 25°C–300°C. At 300–400°C, the δ^13^C value of charcoal decreases slightly, and C = C bonds formed at 400–500°C. At this stage, the change of δ^13^C value depends on the redox reaction process. Oxidation reactions occur when C-H bonds are broken during the bonding process, and the δ^13^C of charcoal decreases; reduction reactions occur when C-O bonds are broken, and the δ^13^C of charcoal increases [46,52,53]. Thus, the δ^13^C values of *Pinus armandii*, *Pinus massoniana*, *Quercus acutissima Carr*, *Quercus aliena Blume*, and *Quercus dentate Thunb* increased with carbonization temperature, and the δ^13^C values of *Pinus tabulaeformis*, *Quercus aliena Blume* var., and *Quercus mongolica Fischer* decreased with temperature; the changes were related to the redox reaction processes during carbonization (Fig 2).

**Fig 3:**
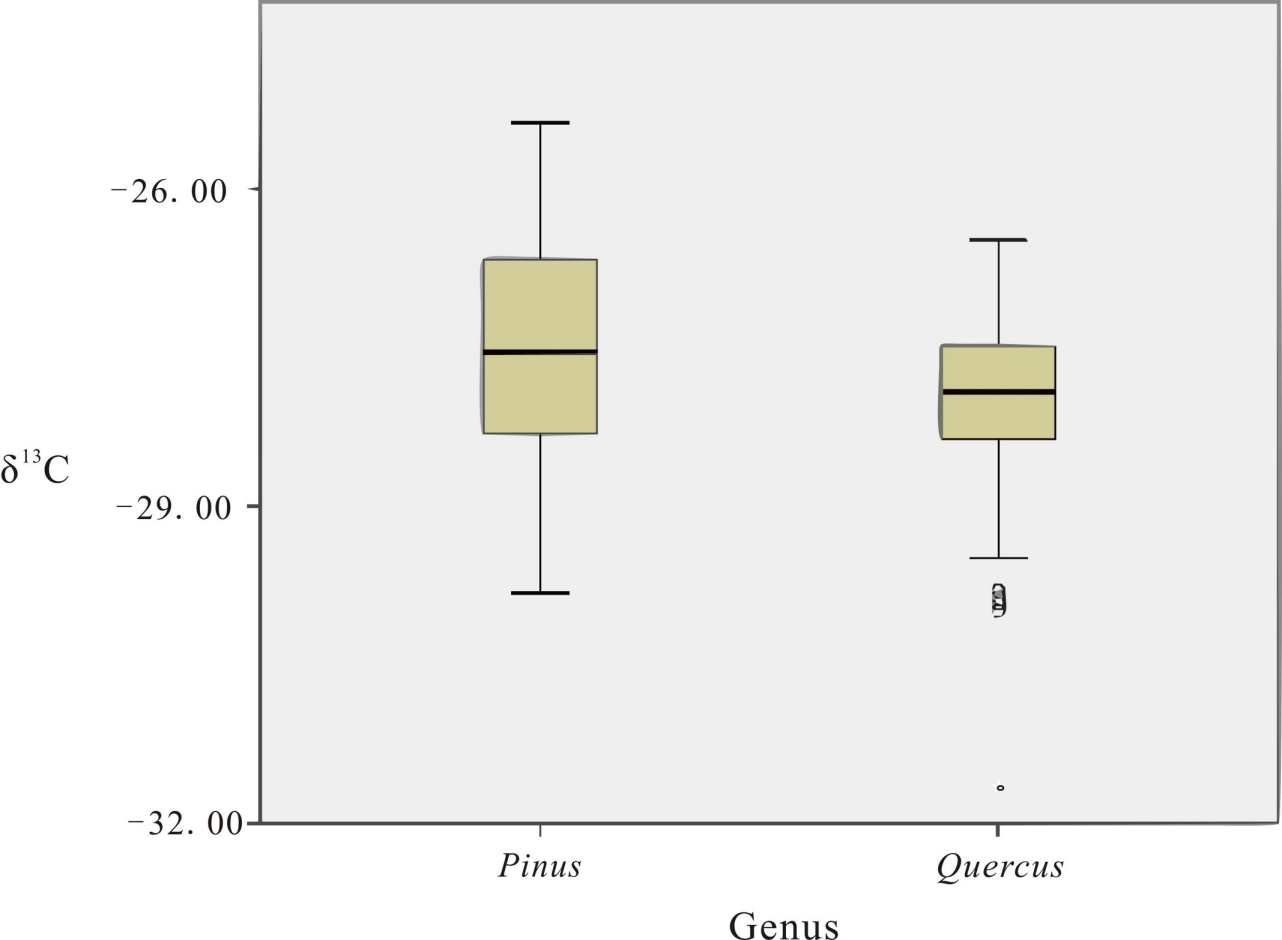
Comparison of δ^13^C (‰ VPDB) data for *Pinus* sp. and *Quercus* sp.

The carbon isotopes of trees are derived from two sources, the plant itself and the fixation of atmospheric CO_2_ by plant photosynthesis [17,31,54,55]. Nonparametric tests for independent samples comparing *Pinus* and *Quercus* genera under the same climatic conditions showed significance(sig) < 0.05(Table 3) for Caobazi, Zhenba, Fengyukou, and Zhidan, which in summary shows that there is a significant difference between *Pinus* sp. and *Quercus* sp. samples in general. Comparing the *Pinus* and *Quercus* genera under the same carbonization conditions, it is obvious that the values of *Pinus* genera are higher than those of *Quercus* genera (Fig 3). The atmospheric CO_2_ was consistent between *Pinus* sp. and *Quercus* sp. under the same growth conditions, and the variability in δ^13^C between the two was caused by the physiological characteristics of the plants; the stomatal conductance of *Pinus* sp. was lower than that of *Quercus* sp., reducing the concentration of intercellular CO_2_ and thus increasing δ^13^C [56]; also, the nitrogen concentration on the leaf surface of *Pinus* sp. was lower than that of *Quercus* sp., leading to a lower efficiency of photosynthesis N concentration on the leaf surface of *Pinus* sp. was lower than that of *Quercus* sp., resulting in lower photosynthetic efficiency than *Quercus* sp. and thus decreasing ^12^C, increasing δ^13^C [57,58]. From the above analysis, there were significant differences in δ^13^C among charcoal genera.

**Table 3:** Nonparametric tests for wood charcoal.

		sig
*Pinus*		0.481
*Quercus*		0.075
*Pinus-Quercus*	Caobazi	0.000
	Fengyukou	0.000
	Zhenba	0.000
	Zhidan	0.023
Wild-indoor carbonization		0.856

The δ^13^C of different genera of charcoal was significantly different, and further comparison of the differences between different species of charcoal of the same genus was performed. Due to the limitations of vegetation distribution, the genus *Pinus* has only one species in Zhidan, Fengyukou, CaoBaZi and JiWoZi and two species in Zhenba; the genus *Quercus* has only one species in Zhidan, Zhenba and CaoBaZi and three species in Fengyukou. Therefore, we will apply the independent sample nonparametric test to analyze the interspecific differences between the genus *Pinus* in Zhenba and the genus *Quercus* in Fengyukou. The results showed a significant value of 0.404 (sig>0.05) between *Pinus* genera and a significant value of 0.298 (sig>0.05) for *Quercus* genera (Table 3), indicating that there were no significant differences between species of *Pinus* genera and between species of *Quercus* genera.

According to our results combined with previous research results [2,59], a significant difference in isotopic fractionation occurs at the genus level, but there is little difference in isotopic fractionation between different species of the same genus, so the isotopic analysis of charcoal can be performed based on the genus level.

### 5.2 Comparison of wildfire carbonization and indoor carbonization

Carbonization of extant wood to charcoal and determination of charcoal stable carbon isotopes is the first step in quantitative paleoclimate reconstruction, which currently uses isotopes to reconstruct paleoclimate by indoor carbonization to obtain δ^13^C of current species and establish a linear relationship [19,26,37], while reconstruction of paleoclimate The samples used are derived from wildfire carbonization, whether these two different carbonization methods affect charcoal stable carbon isotope fractionation and determine whether the relationship model established for extant species can be applied to paleoclimate reconstruction, and thus, both methods need to be further explored.

In this paper, wildfire carbonization and indoor carbonization experiments were conducted with *Pinus* sp. and *Quercus* sp. as test samples, and a nonparametric test was performed on two independent samples of the results obtained from wildfire carbonization and indoor carbonization, and we chose the mean of the carbonization results for the analysis due to the heterogeneity of the wildfire carbonization temperature, which showed sig >0.05 (Table 3). This shows that there is no difference between the δ^13^C obtained by the two carbonization methods. The differences between the two types of carbonization were mainly reflected in three aspects: heating method, carbonization environment and oxygen amount. The samples in this experiment were all wrapped with aluminum foil during carbonization, which acted as an oxygen barrier, so the differences in carbonization environment and oxygen amount were negligible. The heating method of wildfire carbonization is a low to high temperature ramp-up, while the heating method of indoor carbonization is a constant temperature ramp-up. The loss of hemicellulose, cellulose, and lignin components is faster during the ramp-up process, and fractionation is faster under wildfire carbonization than indoor carbonization, but the early fractionation of stable carbon isotopes does not have a major effect on the pyrolysis of the three components [19,35,47,55], it is the final temperature reached in both ways that determines the pyrolysis reaction, and since the three-component has a fixed pyrolysis temperature and content [48], the loss of pyrolytic components such as hemicellulose is the same at the same final temperature of carbonization, and the degree of stable carbon isotope fractionation is the same; therefore, with the same final temperature, the warming and constant temperature processes only affect the rate of pyrolysis reactions of components such as hemicellulose and do not affect the fractionation results of the pyrolysis reactions.

When the carbonization temperature reaches 400°C, the charcoal undergoes redox reactions, and the C-containing chemical bonds inside the wood, including C-O and C-H bonds, break and form C = C bonds. This bonding process affects the fractionation of charcoal δ^13^C. C = C bond formation is faster under constant temperature conditions, and thus, bond formation is faster under indoor carbonization conditions than under wildfire carbonization. Since the composition of chemical bonds is inherent to wood, when wood is completely carbonized, all C-containing bonds are converted to C = C bonds [49,50]. Therefore, for the same genus of wood, warming and constant temperature processes change the rate of redox reactions only during C = C bond formation and do not affect the final bond formation results. In summary, the difference in the carbonization method only changes the rate of fractionation and does not affect the final result of wood pyrolysis fractionation; thus, charcoal obtained by indoor carbonization can be used to simulate wildfire carbonization.

### 5.3 Correlation of the stable carbon isotopic composition of charcoal with climatic factors

The carbon isotope composition in plants is derived mainly from atmospheric CO_2_, Leaf stomata are the main avenue for atmospheric CO_2_ to enter the plant. The stomatal conductance is affected by climate factors such as precipitation (P), evaporation (E), and temperature (T), which affect the fixation of CO_2_ by plants [60–62]. The δ^13^C values of both *Pinus* sp. and *Quercus* sp. wood were significantly negatively correlated with precipitation and positively correlated with evaporation. The relationship between temperature, an important climatic factor affecting plant growth, and plant δ^13^C is more complex. The experimental data also showed that the δ^13^C of *Pinus* sp. was correlated with the mean temperature, while *Quercus* sp. showed no correlation with the mean temperature (Table 4). Carboxylase is a catalyst in plants that uses free HCO^3-^ to act as a catalyst during photosynthesis in the presence of biotin and ATP, and its activity affects the rate of photosynthesis and stomatal conductance, thus changing the plant CO_2_ concentration and affecting plant δ^13^C [60,63]. It was shown that temperature affects the activity of carboxylase, and at temperatures above the optimum temperature range of carboxylase, as the temperature increases, the activity of carboxylase decreases, causing an increase in plant δ^13^C; at temperatures below the optimum temperature range, as the temperature increases, the activity of carboxylase increases, causing a decrease in plant δ^13^C; at temperatures in the optimum temperature range, changes in temperature do not affect the activity of carboxylase, and thus, plant δ^13^C remains unchanged [64–66], while different plants have different temperature adaptations of carboxylase [62,67]; thus, different genera of wood indicators for reconstructing rainfall and evapotranspiration in the study area.

**Table 4:** Pearson correlation coefficients(r) between δ^13^C (‰ VPDB) and climatic parameters of wood.

Genus	δ ^13^C	P(mm)	T(°C)	E(mm)
*Pinus*	δ ^13^C_un_ δ ^13^C_3_ δ ^13^C_4_ δ ^13^C_5_ δ ^13^C_w_	-0.911* -0.937* -0.923* -0.911* -0.768*	-0.584* -0.535* -0.380* -0.553* -0.673*	0.888* 0.878* 0.792* 0.892* 0.832*
*Quercus*	δ ^13^C_un_ δ ^13^C_3_ δ ^13^C_4_ δ ^13^C_5_ δ ^13^C_w_	-0.931* -0.908* -0.888* -0.925* -0.771*	-0.015 -0.008 0.117 0.116 0.326	0.875* 0.880* 0.853* 0.821* 0.890*

* Correlation is significant at the 0.05 level. (P is precipitation; T is temperature; E is evaporation).

**Fig 4:**
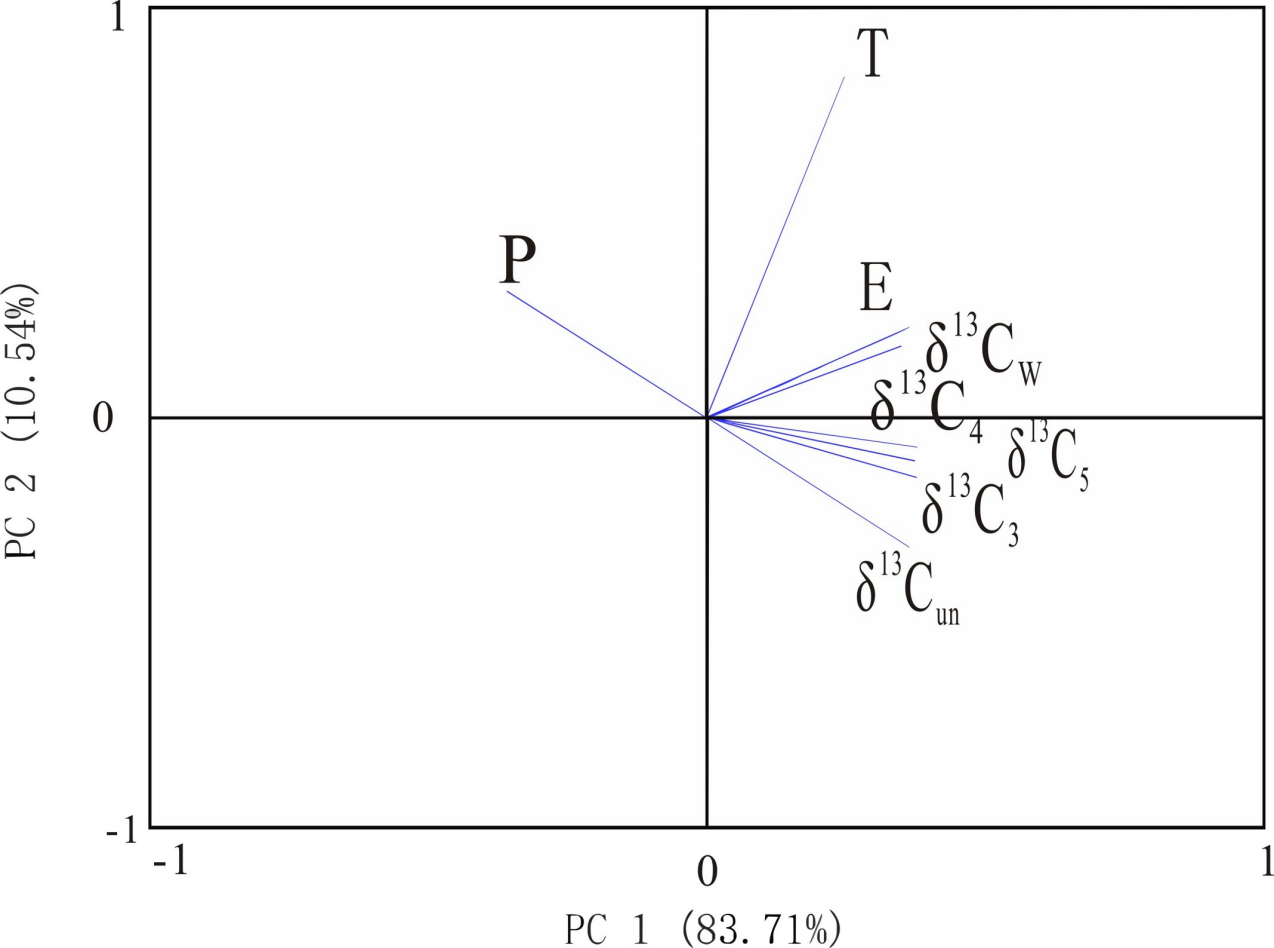
Component weights plot of the principal components analysis (*Pinus*). (P is precipitation; T is temperature; E is evaporation; δ^13^C_un_ (without carbonization); δ^13^C_3_ (300°C carbonization condition), δ^13^C_4_ (400°C carbonization condition), δ^13^C_5_ (500°C carbonization condition), and δ^13^C_w_ (wildfire carbonization condition).

**Fig 5:**
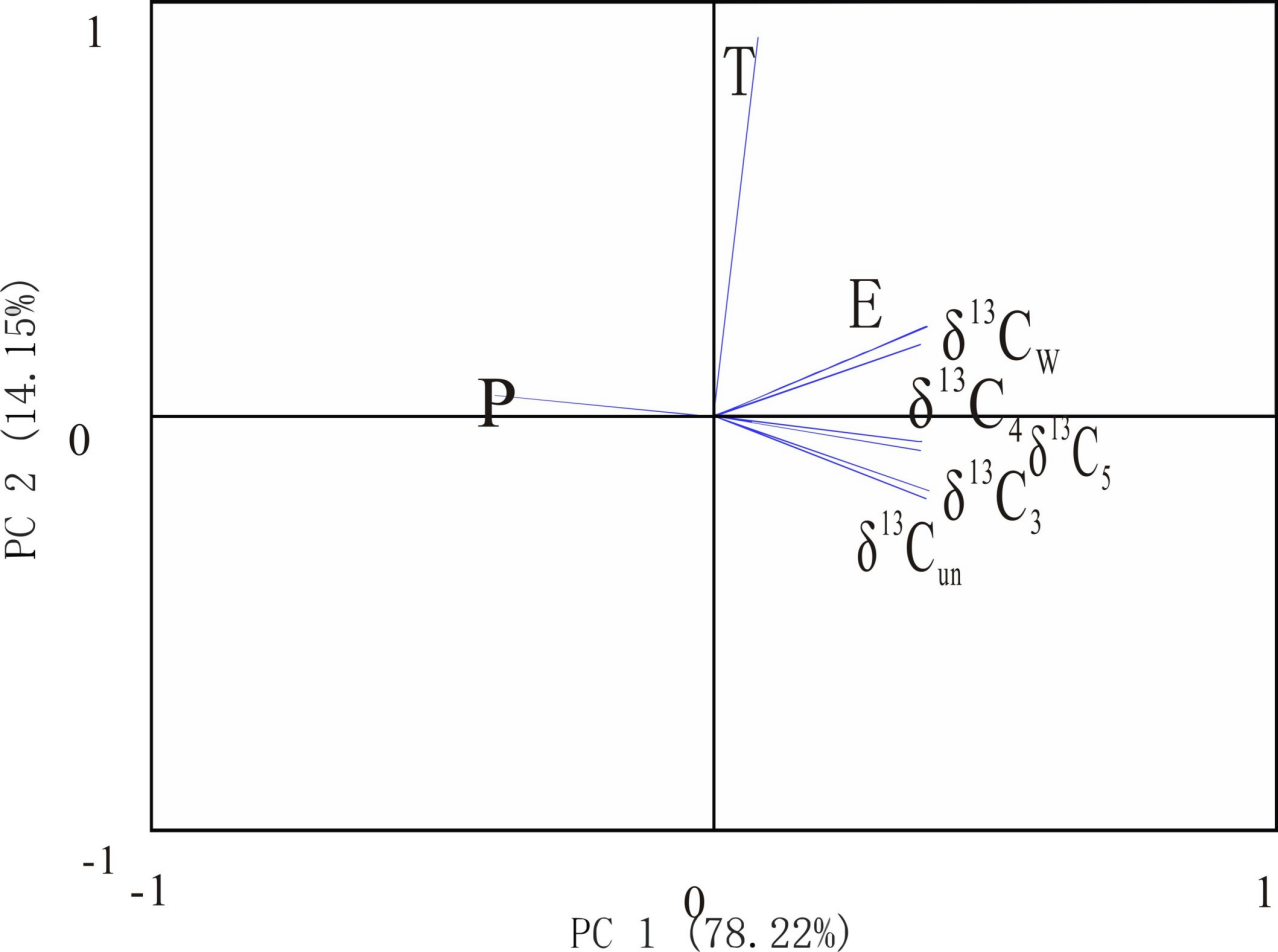
Component weights plot of the principal components analysis (*Quercus*). (P is precipitation; T is temperature; E is evaporation; δ^13^C_un_ (without carbonization); δ^13^C_3_ (300°C carbonization condition), δ^13^C_4_ (400°C carbonization condition), δ^13^C_5_ (500°C carbonization condition), and δ^13^C_w_ (wildfire carbonization condition).

The previous study showed the stable carbon isotopic composition of wood undergoes fractionation during carbonization, so does the stable carbon isotopic composition of charcoal still have the above correlation with climate factors? As seen by the PCA weighting diagram (Figs 4 and 5), the δ^13^C of charcoal at different carbonization temperatures (δ^13^C_3_ (300°C carbonization condition), δ^13^C_4_ (400°C carbonization condition), δ^13^C_5_ (500°C carbonization condition), and δ^13^C_w_ (wildfire carbonization condition) are all distributed in the positive axis part of PC1 and distributed along the PC2 axis from the negative axis to the positive axis in the same order as the δ^13^C values from large to the positive axis. The order of distribution along the PC2 axis from the negative axis to the positive axis is generally consistent with the order of δ^13^C values from large to small. In Fig 4, the direction of δ^13^C is opposite to P and T, and the angle with T is greater than P, and it is in the same direction with E, indicating that *Pinus* sp. charcoal is negatively correlated with rainfall and temperature and positively correlated with evaporation; in Fig 5, the direction of δ^13^C is opposite to P, perpendicular to T, and in the same direction with E, indicating that *Quercus* sp. charcoal is negatively correlated with rainfall, positively correlated with evaporation, and has no obvious correlation with temperature relationship, which is consistent with the wood performance (Table 4). Thus, the fractionation of δ^13^C of charcoal during carbonization does not mask relevant climatic information, and in summary, charcoal δ^13^C is a potential indicator of rainfall and evaporation in the reconstructed study area.

**Fig 6:**
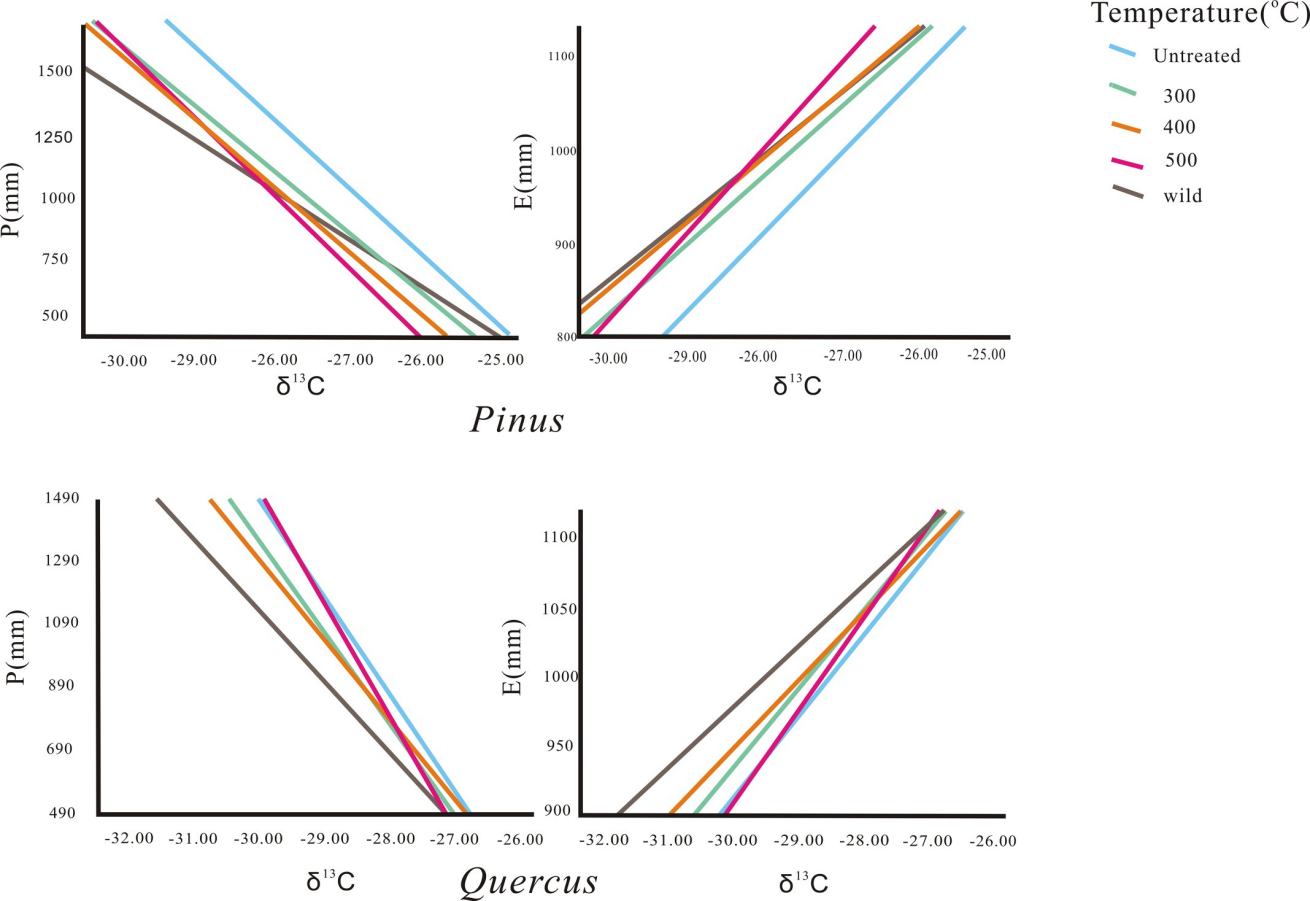
Relationship between the δ ^13^C (‰ VPDB) value and climate factor.

Linear regression analysis of δ^13^C of charcoal of *Pinus* sp. and *Quercus* sp. with rainfall and evaporation at the sampling sites (Table 5) showed good linearity in the fit between charcoal and climatic factors at different temperatures (Fig 6). In the future, charcoal samples of *Pinus* sp. and *Quercus* sp. obtained from archaeological sites can have their δ^13^C values determined, and the carbonization temperature can be determined by Raman spectroscopy [31]. Then, substituting the charcoal δ^13^C into the above linear relationships for the corresponding temperatures can reconstruct the paleo-rainfall and paleo-evaporation in the study area. It is worth noting that the use of fossil fuels since the industrial revolution has led to a significant increase in atmospheric CO_2_ concentration, and when using the linear relationship, the sample δ^13^C needs to be corrected to remove the effect of changes in atmospheric CO_2_ concentration on carbon isotope fractionation.

**Table 5:** Linear regression analysis of δ^13^C (‰ VPDB) of charcoal (P is precipitation; E is evaporation).

Genus		P(mm)	R^2^	E(mm)	R^2^
*Pinus*	untreated	P = -7110-302 δ^13^C/‰	0.831	E = 3290+85.25 δ^13^C/‰	0.789
	300°C	P = -6530-274 δ^13^C/‰	0.877	E = 3040+74.27 δ^13^C/‰	0771
	400°C	P = -6900-285 δ^13^C/‰	0.851	E = 2960+70.67 δ^13^C/‰	0.627
	500°C	P = -8020-324δ^13^C/‰	0.829	E = 3560+91.69 δ^13^C/‰	0.795
	wild	P = -4970-215 δ^13^C/‰	0.590	E = 2870+67.28 δ^13^C/‰	0.691
*Quercus*	untreated	P = -7670-306 δ^13^C/‰	0.866	E = 2660+58.48 δ^13^C/‰	0.765
	300°C	P = -7220-287 δ^13^C/‰	0.824	E = 2630+56.48 δ^13^C/‰	0.774
	400°C	P = -6270-253 δ^13^C/‰	0.813	E = 2410+48.7 δ^13^C/‰	0.728
	500°C	P = -8960-350 δ^13^C/‰	0.788	E = 2880+65.81 δ^13^C/‰	0.675
	wild	P = -5540-224 δ^13^C/‰	0.856	E = 2290+43.68 δ^13^C/‰	0.792

## 6. Conclusions

Significant differences in isotopic fractionation existed at the genus level, while the differences in isotopic fractionation between species of the same genus were not significant, so the charcoal could be analyzed isotopically based on the genus level.

There is no significant difference between the δ^13^C of charcoal obtained by wildfire carbonization and indoor carbonization. The δ^13^C of charcoal of a certain genus can be obtained based on the simulation of indoor carbonization, and a linear relationship between δ^13^C and the climate factor of charcoal can be established.

After wood is carbonized to charcoal by burning, the original climate information is still preserved, and charcoal δ^13^C is a potential indicator for reconstructing rainfall and evaporation in the study area. The δ^13^C values of charcoal samples obtained from archaeological sites can be used to reconstruct paleorainfall and paleoevaporation after correcting for the effect of atmospheric CO_2_ changes on them.

## Supporting information

S1 Table Raw data for all analyses. (XLSX)
